# Preference for facial averageness: Evidence for a common mechanism in human and macaque infants

**DOI:** 10.1038/srep46303

**Published:** 2017-04-13

**Authors:** Fabrice Damon, David Méary, Paul C. Quinn, Kang Lee, Elizabeth A. Simpson, Annika Paukner, Stephen J. Suomi, Olivier Pascalis

**Affiliations:** 1 Univ. Grenoble-Alpes, LPNC, France; 2 CNRS, LPNC,UMR 5105, France; 3 University of Delaware, USA; 4 University of Toronto, Canada; 5 University of Miami, USA; 6 Eunice Kennedy Shriver National Institute of Child Health and Human Development, USA

## Abstract

Human adults and infants show a preference for average faces, which could stem from a general processing mechanism and may be shared among primates. However, little is known about preference for facial averageness in monkeys. We used a comparative developmental approach and eye-tracking methodology to assess visual attention in human and macaque infants to faces naturally varying in their distance from a prototypical face. In Experiment 1, we examined the preference for faces relatively close to or far from the prototype in 12-month-old human infants with human adult female faces. Infants preferred faces closer to the average than faces farther from it. In Experiment 2, we measured the looking time of 3-month-old rhesus macaques (*Macaca mulatta*) viewing macaque faces varying in their distance from the prototype. Like human infants, macaque infants looked longer to faces closer to the average. In Experiments 3 and 4, both species were presented with unfamiliar categories of faces (i.e., macaque infants tested with adult macaque faces; human infants and adults tested with infant macaque faces) and showed no prototype preferences, suggesting that the prototypicality effect is experience-dependent. Overall, the findings suggest a common processing mechanism across species, leading to averageness preferences in primates.

## Introduction

Face processing is of major importance for primates living in large and complex social networks, and plays a crucial role in the formation of inter-individual relationships with multiple group members. Given the importance of face perception for primates, it is reasonable to suggest that such a cognitive skill would be subject to selective pressure through the course of evolution^1^, and it has been argued that there may be a common primate face recognition system^2^. Accordingly, several aspects of face processing are shared between humans and nonhuman primates throughout ontogeny and phylogeny^3^,^4^. For example, at a behavioral level, the distribution of eye movements in humans and macaques during face scanning appears relatively similar, showing the same systematic modulations with stimulus manipulations such as blurring or inversion^5^. Moreover, humans fixate more on the eyes than on any other facial feature^5^,^6^, which is also consistently reported in adult macaques^5^,^7^,^8^,^9^,^10^ and infants^11^,^12^,^13^,^14^.

At a neural level, examination of the organization of face-selective regions across the temporal lobe in both humans and macaques has revealed a close anatomical correspondence between the human and macaque face-processing systems^2^,^15^,^16^. Moreover, macaque face processing has been shown to be norm-based (i.e., prototype-based) at the cellular level^17^. Using single-cell recordings, it has been reported that face-responsive neurons of the anterior inferotemporal cortex of adult macaque monkeys show a tuning centered around the average face. These neurons monotonically increased firing for increased levels of caricaturization (i.e., exaggeration of distinguishing features) for a previously learned face. This result suggests that face-processing in macaque monkeys is organized around a prototypical face representation. A similar finding was reported in humans, showing that adaptation to a face biased the perception of subsequently presented faces along an identity trajectory away from the adapting face, and along an axis centered on the prototypical face representation^18^.

Similarities between humans and macaques have also been reported for early developmental processes. Infant macaques present sensitivity for basic face structures (i.e., first-order relations, eyes above the nose, nose above the mouth). They prefer stimuli that respect face-like configuration^19^,^20^,^21^, and prior work has revealed a similar sensitivity in human neonates^22^,^23^. Later, between 3 and 12 months of age, human infants exhibit a perceptual tuning for faces in a way that makes processing and recognizing infrequently seen faces (e.g., monkey faces) more difficult^24^, a phenomenon likely driven by experience^25^,^26^,^27^. A similar specialization has been shown in Japanese macaques^28^, whose face viewing preference and discrimination performance was biased towards the species category of faces to which the macaques were first exposed, following a total face deprivation period of up to 6 months^28^. Despite the aforementioned commonalities, species-specificities in face processing should not be negated^4^, and a number of issues remain unaddressed. In particular, while humans show a robust preference for facial averageness (i.e., the proximity of a face to the average of a population, in terms of mathematical trait values), it is unknown whether non-human primates are sensitive to such characteristics^3^.

In humans, computer generated composite faces, or prototypes, are appealing to adults^29^,^30^, and 6-month-olds look longer at composite faces than at faces rated as unattractive by adults^31^. Furthermore, typical faces are judged more attractive than distinctive faces^32^,^33^, and a meta-analysis has shown that the appeal of averageness is not restricted to face race or sex^34^, although it might be linked with visual experience^35^.

Two non-mutually exclusive frameworks have been put forward to explain the attraction to average faces in humans. Preferences for attractive faces might have been shaped by sexual selection pressures, as an adaptation for mate choice, because attractiveness and its components may serve as indicators of mate quality, such as health or parasitic resistance^36^,^37^, but see ref. 38. Average traits in a face are linked with greater genetic diversity which may result in greater parasitic resistance^37^, whereas deviation from average could signal chromosomal disorders^39^, at least for lower scores of face prototypicality (i.e., the “bad genes” hypothesis^40^).

The preference for averageness may reflect a side-effect of sensory processing and may be a by-product of the way brains process information in human adults^34^,^41^,^42^,^43^. That is, prototypical stimuli are more fluently processed and human adults show a robust preference for fluently processed stimuli^44^,^45^,^46^. As would be expected given the preferences, fluently processed stimuli elicit positive affect^47^,^48^. Prototypical stimuli are not only fluent, but also feel subjectively familiar^49^,^50^, and familiar stimuli tend to be treated positively (e.g., ref. 51), especially when it comes to faces^52^. The link between familiarity and fluency even goes further, since a feeling of familiarity can arise from the fluent processing of a novel stimulus, being prototypical^53^ or not^54^,^55^,^56^. In this framework, preferences for averageness could be understood as by-products of memory and perception.

Although these two accounts could be seen as alternatives, they are both biologically rooted, and each may have been shaped by evolutionary pressures^34^,^43^. Moreover, even a sensory bias that leads to a general preference for average stimuli would incidentally lead to adaptive choices if averageness is a true signal of health in some domains (e.g., for mate or food selection). Following that rationale, it has been suggested that an initial sensory bias for prototypicality later evolved as a signal of mate quality^34^,^57^. In that case, the general prototypicality bias would phylogenetically predate the signal of mate quality, and might therefore be present in other species sharing a common ancestor with humans, such as nonhuman primates. Such species might share this by-product bias or preference for averageness, even if averageness is not a signal of mate quality in the species considered.

Indirect evidence suggesting a prototypicality effect in nonhuman primates is plausible. For example, adult and infant rhesus macaques show own-species preferences^58^,^59^, but see ref. 60. In a face-space model^61^, own-species faces are clustered closer to the center of the space, whereas other-species faces are clustered further away from the center. Own-species faces can thus arguably be considered as more prototypical than other-species faces. Following this reasoning, part of the preference for own-species faces could stem from a preference for prototypical stimuli. Also, the direction of preferences for faces in infant Japanese macaques has been shown to be highly dependent on visual experience^28^. Infant monkeys exposed only to monkey faces showed preferences for monkey faces, whereas infant monkeys exposed only to human faces showed preferences for human faces; both groups showed a preference for familiar faces. If prototypical faces are found to be more familiar, they might be preferred to other less prototypical faces.

The purpose of the current study was to test whether facial averageness is preferred in human and rhesus macaque infants. In a population, individual faces naturally deviate from the central tendency: some are closer to the prototype, and others are farther away. If infants are sensitive to prototypicality, they might display visual preferences for faces closer to the prototype compared to faces farther from it. However, in the human infant face perception literature to date, previous work experimentally manipulating facial averageness has been inconclusive^62^ or has even revealed preferences for faces manipulated to be non-average^63^. However, it has also been reported that human infants show a preference for prototype faces (computer generated faces) over individual unattractive faces^31^. It is therefore unclear whether such preferences might also be found for typical natural individual faces (faces not computer generated) in human infants. Moreover, data on face averageness preferences in infant macaques are nonexistent^3^. A comparative developmental approach is appropriate for addressing this gap in the literature as infants are relatively free from cultural standards of aesthetics, which otherwise could be conflated with the preference for averageness. Besides, if the prototypicality effect is a by-product of brain processing, it should already be present in relatively “naïve” cognitive systems, i.e., those possessed by infants, provided that the infants have had some experience with the category of stimuli presented.

## Experiment 1

In Experiment 1, we examined the preference of human infants for human adult female faces closer to or farther from an average prototype face.

### Method

The experiment was performed with approval and under the accordance of the relevant guidelines and regulations established by the local ethics committee (“Comité d’éthique des centre d’investigation clinique de l’inter-région Rhône-Alpes-Auvergne”, Institutional Review Board), and informed parental consent was obtained.

#### Participants

Twenty-nine 12-month-old human infants (*M* = 374.52 days, *SD* = 5.54 days, 14 females) participated. Two additional 12-month-olds were excluded due to fussiness.

#### Stimuli and procedure

Stimuli were grayscale images of 32 adult female Caucasian faces, matched for average luminance, contrast, and size. Each image was 405 (width) x 630 (height) pixels. Pictures were placed in an oval shape so that ears, hair, and background were covered. Images were presented against a standardized gray background. Infants were tested in a quiet room and seated in a baby seat away from a 60 cm monitor (Iiyama Vision Master Pro 514, 40 × 30 cm), with a resolution of 1024 × 768 pixels and frame rate of 100 Hz, onto which the images were projected. Parents were seated behind the infant, and were instructed to remain quiet during testing. During the visual preference task, left eye positions of the infants were measured by an EyeLink^®^ 1000 (SR Research Ltd., Mississauga, Ontario, Canada) at a sampling frequency of 250 Hz using the head-free setting with target sticker.

A standard 5-point calibration procedure was conducted before the beginning of each experimental session. From the 32 faces, 4 pairs of faces were selected randomly, separately for each infant, and presented for 5 s after the first look toward one of the faces. Immediately after these four trials and without pause, the same pairs were presented again, with the left/right positions of the faces reversed. In total, infants were presented with 8 trials. Before each trial, an attention-getter attracted infant gaze toward the middle of the screen. We chose not to present the average face because this was a computer generated face, and not a real individual. Thus, if there was a preference for this face it could have been argued that this was due to the unnatural features of the prototype face. Another issue was that if we presented individual faces versus the prototype face, infants would habituate to the average face throughout the trials, thereby interfering with our measure of preference for prototypicality. Furthermore, our interest was in examining averageness preferences among individual faces; therefore, the use of real individual faces varying from the average best suited our objectives.

#### Averageness measure

**Figure 1:**
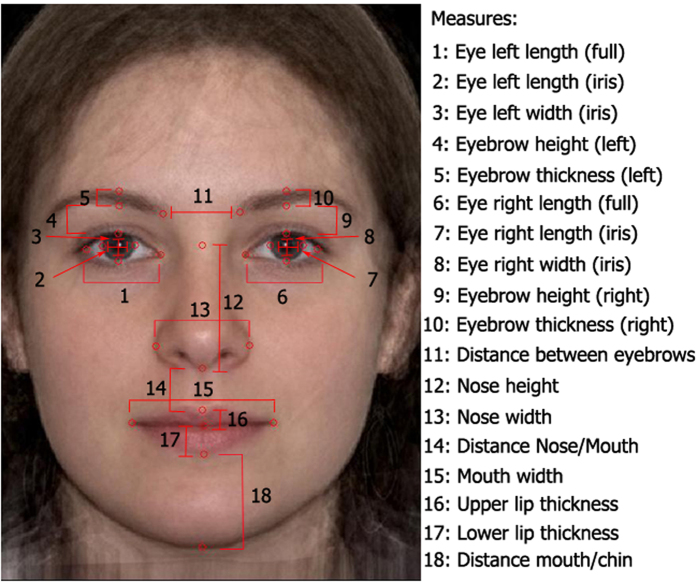
An adult female prototype face with measurement points and measures taken from each of the faces used in Experiment 1. The prototype was not presented to the infants.

The measure of averageness was taken as the distance of the 32 individual faces from a prototype of 42 adult Caucasian female faces (including the 32 faces used in the current experiment). The prototype was constructed using Psychomorph^64^, with a methodology similar to previous face preference studies in infants and adults^30^,^63^,^65^,^66^. Faces were scaled so that all faces were aligned on pupil center. The key locations (52 points) of each of the original individual faces were manually marked, delimiting face features and global shape (e.g., nose, eyes, mouth, jaw line), and then averaged in one prototypical face (see Fig. 1, left). The measure of the distance between the individual faces and the prototype was based on 18 facial measurements (see Fig. 1, right) known to be linked with attractiveness, symmetry, or femininity judgments in face preference tasks conducted with adults^66^,^67^,^68^. Measures were taken on each of the individual faces, subtracted to their corresponding values on the prototype, and then z-score transformed (for further details of the measures, see Supplementary materials). The 18 measures per face were then averaged to obtain the final measure of distance from the prototype. We sought to devise a measure that captured the variation in attractiveness judgments while being solely based on physical distance from the prototype. As an assessment of the validity of our measure, we verified that there was a correlation between the attractiveness judgments made by adult observers on the faces, and our measure of distance from the prototype. Thirty-eight adult raters (*M* = 20.08 years, *SD* = 2.33 days, 28 females) judged the faces on a 5-point scale of attractiveness, with 5 being “very attractive” and 1 being “very unattractive” (*M* = 2.56, *SD* = 0.40). There was a negative correlation between the attractiveness ratings and the distance from the prototype measure, *r*(30) = −0.49, *p* = 0.005, 95% CI [0.17, 0.72], indicating that decrease in distance from the prototype resulted in higher scores in attractiveness ratings, consistent with previous work linking prototypicality and attractiveness ratings^33^,^69^.

### Results and Discussion

Trials were excluded if the infant failed to look at both stimuli. The mean number of remaining trials per infant after exclusion was 7.10 (*SD* = 1.15). The area of interest was the entire face, including the outer contour, i.e., 405 (width) x 630 (height) pixels. Only fixations measured in this area were included in the analysis. All calculations for the identification of fixations were done in degrees of visual angle. The parsing of the eye data into saccades and fixations was performed in accord with previously reported methods^70^. Parameters were adjusted for saccade detection. The fixations within each trial were then classified according to their locations (on left or right face, or elsewhere on the screen). For each participant, we calculated the total looking time, the number of fixations, and the average individual fixation duration on each face.

Because stimulus pairs were randomly selected from the set of faces, the difference in distance from the prototype between the two faces varied from trial to trial. Within each pair however, one face was relatively closer to the prototype than the other. We therefore categorized, for each trial, faces as “closer” to or “farther” from the prototype, and then averaged all “close” trials together and all “far” trials together, separately for each infant. Infants showed greater total looking time to the faces closer to the prototype compared to the faces farther from the prototype, *t*(28) = 2.79, *p* = 0.009, Cohen’s *d* = 0.52, 95% CI [67, 437],(*M*_*c*los*e*_ = 2364 msec, *SD*_close_ = 354 msec, and M_far_ = 2111 msec, *SD*_far_ = 321 msec). A similar result was found with the number of fixations: there were more fixations on the faces closer to the prototype than on the faces farther from it, *t*(28) = 2.71, *p* = 0.011, Cohen’s *d* = 0.50, 95% CI [0.13, 0.93], (*M*_*c*los*e*_ = 5.76, *SD*_close_ = 1.28, and *M*_far_ = 5.23, *SD*_*far*_ = 1.08). Mean fixation duration was nominally greater on faces closer to the prototype than on faces farther from it, but the difference was not significant, *t*(205) = 1.30, *p* = 0.200, Cohen’s *d* = 0.09 95% CI [−12, 58], (*M*_clos*e*_ = 459 msec, *SD*_close_ = 254 msec, *M*_far_ = 436 msec, *SD*_far_ = 174 msec).

Stimulus analysis revealed a significant negative correlation between the looking time on faces and the measure of distance from the prototype, *r*(30) = −0.52, *p* = 0.002, 95% CI [−0.73, −0.21]. It is worth adding that the adult attractiveness ratings described in the Method section were a good predictor of infant looking time. Infants looked longer at the faces rated relatively more attractive compared to the less attractive faces, *t*(28) = 2.86, *p* = 0.008, Cohen’s *d* = 0.53, 95% CI [10, 582], (M_more_ = 2407 msec, *SD*_more_ = 435 msec, and *M*_less_ = 2068 msec, *SD*_less_ = 353 msec). There was also a correlation between the looking time at faces and the attractiveness ratings, *r*(30) = 0.46, *p* = 0.008, 95% CI [0.13, 0.70], indicating that higher attractiveness ratings were associated with longer looking times. The results extend findings showing that 6-month-old human infants prefer a computer generated prototypical face compared to faces rated as unattractive^31^ by revealing the same type of effect in older infants using individual faces naturally varying around an average.

## Experiment 2

In Experiment 2, we examined the preference of macaque infants for infant macaque faces closer to or farther from an average face.

### Method

The following procedures were approved by the *Eunice Kennedy Shriver* National Institute of Child Health and Human Development Animal Care and Use Committee. The study was conducted in accordance with the Guide for the Care and Use of Laboratory Animals and complied with the Animal Welfare Act.

#### Participants

Twenty-six rhesus macaque infants (*Macaca mulatta*), were tested at 3 months of age (15 females*, M* = 95.5 days, *SD* = 3.5 days). Macaque infants were tested at this age to approximately match the 12-month-old human infants in terms of visual development, following a 4-to-1 developmental timetable^71^. Three infants were excluded due to fussiness (*n* = 1), or because they were detected as outliers (*n* = 2) based on the median absolute deviation (i.e., median plus or minus 2.5 times median absolute deviation, see ref. 72). Infants were healthy and were separated from their mothers on the first day of life, after which they were reared in a nursery facility for unrelated studies. Infants were individually housed in incubators (51 cm × 38 cm × 43 cm) for the first two weeks of life and in cages thereafter. Both housing arrangements contained a fleece surrogate and toys, and gave infants constant visual access to same-age conspecifics. From days 1–36 of life, infants had constant visual exposure to same-age conspecific faces from other infants housed in adjacent cages. They could see and hear, but not physically contact other infants. From 37 days of age, half the infants were housed in small groups and half were housed individually but received 2 hours per weekday of playtime with peers, so all infants had visual experience with conspecifics (for more details about rearing, see ref. 73). Human caregivers wore personal protective equipment, including goggles, masks covering the nose and mouth, and hats, so only their eyes were visible (see ref. 74). The face experience of the infant monkeys was thus extremely well controlled; they were exposed only to peer faces of similar ages to themselves, and had no experience with adult monkey faces.

#### Stimuli and procedure

Stimuli were 8 pairs of grayscale images of infant monkey faces (aged from 30 to 90 days) taken from a previous cohort at the Laboratory of Comparative Ethology and unfamiliar to the infants. The 8 pairs of faces were randomly created at the beginning of the experiment and then systematically used for all infants. Infant stimulus faces were chosen because the infant participants had experience with infant monkey faces (see rearing practices, in the Participants section). Each infant macaque face was 380 (width) x 480 (height) pixels. Images were matched for average luminance, contrast, and size. Pictures were placed in an oval shape so that ears, hair, and background were covered. Stimulus preparation followed the same procedure as in Experiment 1. One experimenter held the infants 60 cm from the screen. Infants either clung to the experimenter or were held in a fleece pouch. Each infant was calibrated using a 5-point calibration to Tobii Studio’s pre-set locations.

Infants viewed four face pairs in each test session, and completed two test sessions on two separate days. A central cartoon and music attracted infant attention to the center of the screen, at which time the experimenter pressed a key to start the first trial. The pairs of faces were shown for 10 s, after which the attention-getter appeared again until the infant fixated on the screen, at which point the next trial started. Infant eye movements were recorded via corneal reflection using a Tobii TX300 eye tracker, a remote 58.4 cm monitor with integrated eye tracking technology, and a sampling rate of 60 Hz. We used Tobii Studio software (Tobii Technology, Sweden) to collect the data.

#### Distance measure

**Figure 2:**
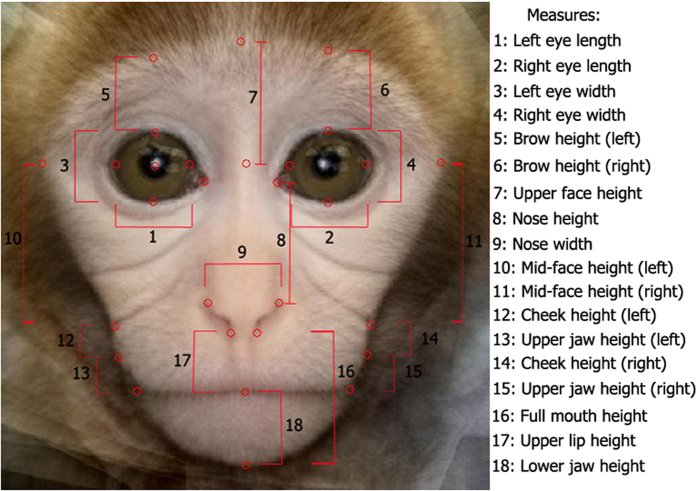
Macaque infant prototype face with measurement points and measures taken from each of the faces used in Experiments 2 and 4. The prototype was not presented to the infants.

We computed distance measures for each of the 16 individual faces from the prototype. The prototype was the average of the 16 infant rhesus monkey faces used in Experiment 2 (see Fig. 2), constructed as in Experiment 1. The measure of the distance between the individual faces and the prototype was computed based on the facial measurements used in previous studies in rhesus macaques^75^.

### Results and Discussion

Data analysis was similar to that in Experiment 1. Only trials where participants gazed at both faces were included in the dataset. The mean number of remaining trials per infant after exclusion was 5.86 (*SD* = 1.42). The total looking time, the number of fixations, and the average individual fixation duration on each face were calculated for each participant. The area of interest was the entire face, including the outer contour, i.e., 420 (width) x 560 (height) pixels. Only fixations measured in this area were included in the analysis. Because the 8 pairs of faces presented were fixed and did not vary between participants, mean looking time for faces close to the prototype was computed from the eight faces closer to the prototype and likewise the mean looking time for faces farther from the prototype was computed from the eight faces farther from the prototype. Due to a non-normal distribution (Kolmogorov-Smirnov test, *p* = 0.001), all data were log-transformed prior to analysis.

Macaque infants showed significantly longer total looking time for faces closer to the prototype compared to faces farther from the prototype, *t*(22) = 4.16, *p* < 0.001, 95% CI [0.115, 0.343], Cohen’s *d* = 0.87, (*M*_close = _1018 msec, *SD*_close_ = 438 msec, and *M*_far_ = 815 msec, *SD*_far_ = 291 msec). Although the mean number of fixations was not different from chance, *t*(22) = 0.80, *p* = 0.434, 95% CI [−0.080, 0.180], (*M*_close_ = 4.83, *SD*_close_ = 1.61, and *M*_far_ = 4.61, *SD*_far_ = 1.59), infants did make longer individual fixations on faces closer to the prototype than on faces farther from it, *t*(134) = 4.02, *p* < 0.001, 95% CI [0.085, 0.250], Cohen’s *d* = 0.35, (*M*_close_ = 218 msec, *SD*_close_ = 135 msec, *M*_far_ = 180 msec, *SD*_far_ = 90 msec). Stimulus analyses did not reveal any significant correlation between total looking time to the faces and the measure of distance from the prototype, *r*(14) = −0.16, *p* = 0.54, 95% CI [−0.61, 0.34].

In humans, the prototypicality bias is dependent on the observer’s experience with the categories of faces presented^35^, although the precise amount of experience necessary to induce the bias is unclear. If this same effect of experience applies to the averageness preference of monkeys, then it should only be observed for familiar categories of faces. In contrast with human infants, exposure to faces in monkey infants can be experimentally controlled (e.g., ref. 28). In Experiment 2, the rhesus sample was exposed to macaque infant faces, but not macaque adult faces. In Experiment 3, we tested the same macaque infants with macaque adult face stimuli. If the prototypicality effect in monkeys is also dependent on past visual experience, infant monkeys should not present any preference for more prototypical, as opposed to less prototypical, adult monkey faces.

## Experiment 3

In Experiment 3, we examined the preference of macaque infants for adult macaque faces closer to or farther from an average face.

### Method

**Figure 3:**
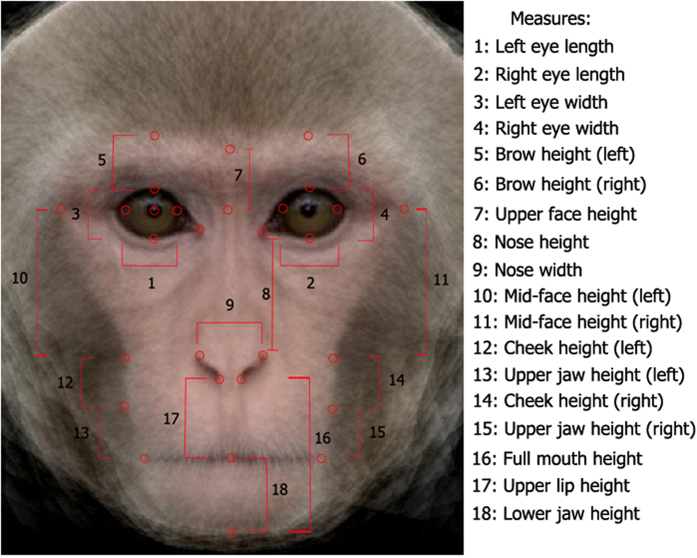
Macaque adult prototype face with measurement points and measures taken from each of the faces used in Experiment 3. The prototype was not presented to the infants.

The following procedures were approved by the *Eunice Kennedy Shriver* National Institute of Child Health and Human Development Animal Care and Use Committee. The study was conducted in accordance with the Guide for the Care and Use of Laboratory Animals and complied with the Animal Welfare Act. Participants and procedure were identical to those in Experiment 2; only the stimuli were different. Stimuli were 8 pairs of grayscale images of adult macaque faces. Each adult macaque face was 420 (width) x 480 (height) pixels. Measures of the distance between the 16 individual faces and the average face were computed following the same procedure as in Experiment 2, but with the macaque adult face stimuli (see Fig. 3). The order of participation in Experiment 2 and Experiment 3 was counterbalanced across infants.

### Results and Discussion

Only trials where participants gazed at both faces were included in the dataset (average per infants: 5.81 trials (*SD* = 1.74). An area of interest surrounded the contour of each of the faces, i.e., 500 (width) x 525 (height) pixels. Only fixations measured in this area were included in the analysis. Because the 8 pairs of faces presented were fixed and did not vary between participants, measures of looking—total looking duration, fixation duration, and fixation frequencies—for faces closer to the prototype were computed from the eight faces closer to the prototype, and likewise, measures of looking for faces farther from the prototype were computed from the eight faces farther from the prototype. Due to a non-normal distribution (Kolmogorov-Smirnov test, *p* = 0.01), all data were log-transformed prior to analysis.

Macaque infants showed no significant preference for faces closer to the prototype compared to faces farther from the prototype, looking equally long to both face types, *t*(25) = 0.04, *p* = 0.97, Cohen’s *d* = 0.01, 95% CI [−0.220, 0.230], (*M*_close_ = 808 msec, *SD*_close_ = 276 msec, and *M*_far_ = 890 msec, *SD*_far_ = 530 msec). The mean number of fixations to both face types did not differ from chance, *t*(25) = −0.43, *p* = 0.67, Cohen’s *d* = 0.21, 95% CI [−0.193, 0.126], (*M*_close_ = 4.24, *SD*_close_ = 1.37, and *M*_far_ = 4.69, *SD*_far_ = 2.54), and neither did the mean fixation duration, *t*(150) = 0.40, *p* = 0.686, Cohen’s *d* = 0.03, 95% CI [−0.071, 0.108], (*M* = 194 msec, *SD* = 97 msec, *M* = 191 msec, *SD* = 95 msec). Moreover, stimulus analysis did not reveal any significant correlation between the total looking time to faces and the measure of distance from the prototype, *r*(14) = 0.43, *p* = 0.09, 95% CI [−0.08, 0.77]. Overall, infant macaques showed no preferences for prototypicality with adult macaque face stimuli.

Taken together, these experiments suggest an experience-dependent prototypicality effect in macaques. If this interpretation is correct, and if evolution maintained the mechanism in humans, then we can predict that 12-month-old human infants should present no prototypicality effect with an unfamiliar category of faces, much like the infant macaques in Experiment 3. In Experiment 4, we therefore tested human infants with infant macaque faces varying in the distance to the prototype. Since a similar prediction can be made with human adults, a control group of 18 adults were also tested with the same procedure and stimuli.

## Experiment 4

In Experiment 4, we examined human infant and adult preferences for infant macaque faces closer to or farther from an average face.

### Method

The experiment was performed with approval and under the accordance of the relevant guidelines and regulations established by the local ethics committee (“Comité d’éthique des centre d’investigation clinique de l’inter-région Rhône-Alpes-Auvergne”, Institutional Review Board), informed parental consent was obtained for infant participants, and informed consent was obtained for adult participants. Procedure and stimuli were identical to those in Experiment 2.

#### Participants

Eighteen human adult (16 females*, M* = 22.80 years, *SD* = 3.91 years), and 16 12-month-old human infants (7 females*, M* = 368.96 days, *SD* = 24.68 days) were tested. One 12-month-old was excluded due to fussiness. Adult participants had no specific history of being familiar with infant rhesus monkey faces although they all had seen adult monkey faces on television documentaries or in books.

### Results and Discussion

Data analysis was similar to that in Experiment 2. Trials were excluded if the participants failed to look at both stimuli. The mean number of remaining trials per infant after exclusion was 7.20 (*SD* = 1.78) and no trials were excluded for adult participants.

Human adults showed no significant preference for faces closer to the prototype compared to faces farther from the prototype, looking equally long to both face types, *t*(17) = 0.26, *p* = 0.79, Cohen’s *d* = 0.06, 95% CI [−191, 246], (*M*_close_ = 2050 msec, *SD*_close_ = 235 msec, and *M*_far_ = 2023 msec, *SD*_far_ = 225 msec). The mean number of fixations was not different from chance, *t*(17) = −0.31, *p* = 0.76, Cohen’s *d* = 0.07, 95% CI [−0.755, 0.560], (*M*_close_ = 7.33, *SD*_close_ = 0.75, and *M*_far_ = 7.42, *SD*_far_ = 1.05), and neither was the mean fixation duration, *t*(143) = 1.70, *p* = 0.091, Cohen’s *d* = 0.14, 95% CI [−1.6, 21], (*M* = 287 msec, *SD* = 62 msec, *M* = 278 msec, *SD* = 47 msec). Moreover, stimulus analysis did not reveal any significant correlation between the total looking time to faces and the measure of distance from the prototype, *r*(14) = −0.02, *p* = 0.95, 95% CI [−0.51, 0.48]. Overall, human adults showed no preference for prototypicality with infant macaque face stimuli.

Similarly, human infants showed no significant preference for faces closer to the prototype compared to faces farther from the prototype, looking equally long to both face types, *t*(14) = 1.05, *p* = 0.31, Cohen’s *d* = 0.27, 95% CI [−93, 271], (*M*_close* = *_1478 msec, *SD*_close_ = 314 msec, and *M*_far_ = 1389 msec, *SD*_far_ = 444 msec). The mean number of fixations was not different from chance, *t*(14) = 1.80, *p* = 0.09, Cohen’s *d* = 0.47, 95% CI [−0.074, 0.860], (*M*_close_ = 3.89, *SD*_close_ = 1.27, and *M*_far_ = 3.50, *SD*_far_ = 1.20), and neither was the mean fixation duration, *t*(107) = −0.28, *p* = 0.78, Cohen’s *d* = 0.03, 95% CI [−59, 44], (*M* = 435 msec, *SD* = 220 msec, *M* = 443 msec, *SD* = 201 msec). Moreover, stimulus analysis did not reveal any significant correlation between the total looking time to faces and the measure of distance from the prototype, *r*(14) = 0.11, *p* = 0.69, 95% CI [−0.41, 0.57]. Overall, human infants showed no preference for prototypicality with infant macaque face stimuli.

## General Discussion

Expanding earlier reports of preferences for average faces^31^ and attractive faces^76^,^77^,^78^ in human infants, we showed that both human and macaque infants attended more to faces closer to the prototype, suggesting that a common processing mechanism leads to averageness preferences in both species.

Similarities in face-processing mechanisms between human and macaque adults were previously pointed out by an electrophysiological single-unit experiment in macaques, showing evidence of norm-based face encoding^17^ and mimicking psychological mechanisms shown in human face perception^18^. Such findings were interpreted as the outcome of a common internal comparative process between the incoming face and an internal prototype. The current findings of preferences for faces closer to the prototype might be regarded as convergent evidence for such a mechanism, in both human and macaque infants. Faces closer to the internal face representation may be perceived as more familiar^49^,^50^, or may be more easily processed^44^,^45^,^46^, and be preferred for these reasons. It should be acknowledged that exemplar-based models of face recognition can also account for these effects^79^,^80^, given that both categories of models predict an influence of a prototypical face representation. However, exemplar models have come under recent criticism in accounting for a broader range of phenomemon^81^,^82^.

In addition, the prototypicality effect appeared when macaque infants were presented with infant faces for which they have visual experience, whereas no preference emerged when adult faces were used. This result suggests that the nursery-raised infant macaques had developed a discrete representation of macaque infant faces (a category of faces present in their environment), distinct from adult macaque faces (a category of faces not present in their environment). An intriguing related question is whether infants systematically compare incoming faces to their ‘infant tuned’ internal prototype, even when the faces do not fall in the infant category.

The present findings can be interpreted as evidence of a common primate face-processing system, but similar outcomes could also be explained by a more general mechanism of object recognition that is not necessarily face-specific. In humans, prototypicality has been shown to predict attractiveness in a variety of reproductively irrelevant stimuli such as eyeglasses or watches^41^,^42^,^43^. In addition, norm-based encoding has not only been reported for faces, but also for abstract shapes, both in humans and macaques^83^,^84^,^85^,^86^, suggesting that prototypicality might refer to a general principle of brain processing, encompassing but not limited to face processing. Thus, monkeys might also be sensitive to prototypicality in other domains besides faces. Such a possibility is consistent with a recent proposition of a biologically inspired model seeking to reintegrate face processing into a general theory of object representation and recognition^80^,^87^. We focused on facial averageness in the current study, as faces are conveniently present in both human and macaque early environments. Examination of the prototypicality effect was therefore possible without intensive training. The issue of domain-specificity is more difficult to address, because it would require that monkeys follow controlled training with various exemplars of non-face objects from the same category (differing by their prototypicality). From a broader point of view, a prototype learning effect could even be a feature of any visual recognition system^34^, and not be limited to primates. However, such an interpretation should be considered tentative since our current findings only involve two primate species, and therefore do not allow us to generalize further.

Interpreting the basis of the prototypicality effect in infants is also tied to the larger discussion of whether averageness preferences reflect an adaptation for mate choice or arise out of general perceptual mechanisms^34^,^41^,^42^,^43^. A preference for averageness stemming from a mate selection signal in humans is unlikely to be present in another species, unless prototypicality also has evolved as a veridical signal of health and mate quality in rhesus macaques. To date, however, there is no research to support this speculation. Another possibility is to consider whether the preference may have served as a signal of mate quality and, coincidentally, emerged as a side effect of a different psychological mechanism^43^. Such an account, however, is less parsimonious because it calls for two hypotheses in order to explain the same behavior. In the absence of data concerning monkey reliance on face prototypicality to assess health in conspecifics, a more circumspect explanation would be to consider that there is a common primate sensory bias toward prototypicality, which evolved in humans as a mate quality signal. Such an interpretation would make the prototypicality effect a homologous mechanism between humans and macaques. Distinguishing a homology (as a mechanism inherited from a common ancestor) from a homoplasy (as a convergent evolution of similar features evolved independently in different species to cope with the same computational problem) is not straightforward^88^, especially for psychological mechanisms or behaviors that are not accessible in extinct species^89^, compared to skeletal structure, for example. However, resolving such an issue is beyond the scope of the present paper.

In the current study, infant macaques had a controlled face experience as they were exposed to same-aged peers only (i.e. nursery-reared infants), which allowed us to examine the influence of experience in the development of the prototypicality effect. A downside of this controlled exposure is that the perceptual experience of nursery-reared macaques is more limited compared to other macaque infants raised with adult macaques (i.e., mother-reared infants), and could thus be regarded as atypical. However, our findings are consistent with a number of previous studies reporting efficient face processing for own-species faces in nursery-reared infant macaques^13^,^20^,^21^,^74^. Remarkably, these findings are also similar to the patterns observed in human infant own-species biases^27^, suggesting that even infants with limited face exposure to conspecifics still exhibit strong biases to attend to and efficiently process own-species faces. While additional studies with mother-reared infants are needed to determine generalizability, the pattern of data observed in the current and prior studies suggest that there are early and robust own-species preferences in infant macaques, even with atypical/limited conspecific face exposure.

Our findings differ from results of a study that reported a switch from an own-species preferences toward an other-species preferences in mother-reared infant macaques^60^. However, we believe there are methodological differences between the current and prior studies, besides infant rearing, that may account for the differences in preference. For example, in the present study conspecific and heterospecific faces were not shown concurrently; macaque faces were always paired with another macaque face, and human faces were always paired with another human face. By contrast, in the prior study, a conspecific and heterospecific face were shown side-by-side to directly measure preference. If the method of the prior study was used with the type of nursery-reared infants investigated in the present study, it is possible that a switchover from own-species preference to other-species preference would be observed, but future work is necessary to test this possibility.

While the direction of the effect was consistent across both species, the strength of the effect was different. Human infants presented a more consistent preference than macaque infants for averageness, with the effect reflected on both measures of looking time and number of fixations, and also in stimulus analysis. In sum, human infants looked longer and explored more thoroughly faces closer to the prototype. By contrast, macaque infants looked longer at faces closer to the prototype, but did not make more fixations on these faces. Possibly reflecting a trading relation, their fixations were of longer duration on faces closer to the prototype compared to faces farther from the prototype. Longer fixations could indicate a greater interest and deeper level of information processing^11^.

It is not entirely clear what could have induced this differential behavioral pattern between human and monkey infants. One possibility is that humans and monkeys have evolved different face-processing perceptual strategies. Even though the rhesus macaque appears to be a good model for humans in neuroscience, humans and macaques are separated by 25 million years of evolution, thus differences are likely. For example, human infants might develop a more robust representation of individual identity compared to macaque infants^4^. Hence, the former present a stronger prototypicality effect than the latter. Another possibility is that the difference in outcomes might result more from a difference in the amount of experience with faces rather than a real species difference, because 12-month-old human infants had more experience with faces than 3-month-old macaque infants (in terms of amount of time). Consequently, the face representation developed by human infants might be more structured than the one developed by the infant macaques, and their face processing might have become more efficient. Matching participants on visual and cognitive development seemed more critical than matching them on experience – given that doing both, even if ideal, was not possible.

In conclusion, we found behavioral evidence in human and macaque infants for a preference for faces closer to a prototype. The findings are likely linked to visual experience with faces. This study is but a step along the way to establish the existence of a common primate prototypicality effect, and future studies should examine the presence of the effect for nonhuman primates in domains other than faces, as well as in other nonhuman primate species.

## Additional Information

**How to cite this article:** Damon, F. *et al*. Preference for facial averageness: Evidence for a common mechanism in human and macaque infants. *Sci. Rep.* **7**, 46303; doi: 10.1038/srep46303 (2017).

**Publisher's note:** Springer Nature remains neutral with regard to jurisdictional claims in published maps and institutional affiliations.

## Supplementary Material

Supplementary Information

Supplementary Dataset 1
