# Supplementary material for: Preference for facial averageness: Evidence for a common mechanism in human and macaque infants
Source: Sci Rep. 2017 Apr 13;7:46303. doi: 10.1038/srep46303 (PMC5390246; doi:10.1038/srep46303)
Supplement: Supplementary Information [file srep46303-s1.pdf]

Perception of facial averageness:

Evidence for a common mechanism in human and macaque infants

Fabrice Damon[1], David Méary[1], Paul C. Quinn[2], Kang Lee[3], Elizabeth A. Simpson[4], Annika Paukner[5], Stephen J. Suomi[5] & Olivier Pascalis[1]

[1]Université Grenoble-Alpes, France;

[2]University of Delaware, USA;

[3]University of Toronto, Canada;

[4]University of Miami, USA;

[5]Eunice Kennedy Shriver National Institute of Child Health and Human Development, USA.

```
%%to make it work:
%%place the Data.xls file in the same folder than the current script.
%%script to run the analyze from:
%%Perception of facial averageness: Evidence for a common mechanism in
%%human and macaque infants
%%%Experiment 1

clc;clear all;close all;
pathim='.\';
%%Experiment 2:
Res = xlsread('Data.xls','DataHumanExp1','A2:L233');
dist= xlsread('Data.xls','DistanceHumanExp1','A2:B33');

dist=dist';

%%Preprocess: outlier and excuded trials

%remove trials with o msec of looking time on one side
 Filt1=find((Res(:,5))==0);Res(Filt1,:)=[];
  Filt2=find((Res(:,6))==0);Res(Filt2,:)=[];
%%look at the data
 %hist((Res(:,5)-Res(:,6)),20)
 %z score of LT to perfrom KSTEST (normality test)

Norm=(((Res(:,5)-Res(:,6))-(mean((Res(:,5)-Res(:,6)))))/(std(Res(:,5)-Res(:
,6))));
[H,P,ksstat] = kstest(Norm)

 %%Normality is Ok here then no need to log normal transformation
%  Res=[Res(:,1:12) log(Res(:,5)) log(Res(:,6))...
%   Res(:,9) Res(:,10)];
%%to check the number of trial per subject,
Restemp=Res(:,1);
T=unique(Restemp);
 for su = 1:size(T,1)
      TestTrial=find(Restemp(:,1)==T(su));
      if isempty(TestTrial)
      else
           Nb(su)=sum([TestTrial./TestTrial]);
      end
 end
      mean(Nb)
      std(Nb)
 %%Now analyse the data
%calculate the difference between time and between dist
for k=1:size(Res,1)
    DiffTime=(Res(k,5)-(Res(k,6)));
    DiffDist=(Res(k,7)-(Res(k,8)));
    ResProc(k,:)=[Res(k,:) DiffTime DiffDist];
end
%%per subject:
for i = 1:size(ResProc,1)
    if ResProc(i,14)<0
        ResProcAdd(i,:)=[ResProc(i,:) ResProc(i,5) ResProc(i,6)
```

```
ResProc(i,7) ResProc(i,8) (ResProc(i,7)-ResProc(i,8))];
    else
        ResProcAdd(i,:)=[ResProc(i,:) ResProc(i,6) ResProc(i,5)
ResProc(i,8) ResProc(i,7) (ResProc(i,8)-ResProc(i,7))];
    end
end
G=unique(ResProcAdd(:,1));
for i=1:size(G,1)
    Line=find(ResProcAdd(:,1)==G(i));
    G1=ResProcAdd(Line,:);
    meanSub(i,:)=[G(i) mean(G1(:,15)) mean(G1(:,16))];
end
mean(meanSub(:,2))
mean(meanSub(:,3))
std(meanSub(:,2))
std(meanSub(:,3))
[meanSubTest,P,CI,STATS] = ttest((meanSub(:,2)-meanSub(:,3)))
CohenDSub = mean(meanSub(:,2)-meanSub(:,3))/STATS.sd
% to calculate the difference in terms odf fixation number
for i = 1:size(ResProcAdd,1)
    if ResProcAdd(i,14)<0
        ResProcFix(i,:)=[ResProcAdd(i,:) ResProcAdd(i,9) ResProcAdd(i,10)
ResProcAdd(i,11) ResProcAdd(i,12)];
    else
        ResProcFix(i,:)=[ResProcAdd(i,:) ResProcAdd(i,10) ResProcAdd(i,9)
ResProcAdd(i,12) ResProcAdd(i,11)];
    end
end
H=unique(ResProcFix(:,1));
for i=1:size(H,1)
    Line=find(ResProcFix(:,1)==H(i));
    H1=ResProcFix(Line,:);
    meanSubFix(i,:)=[H(i) mean(H1(:,20)) mean(H1(:,21)) mean(H1(:,22))
mean(H1(:,23))];
end
%meanSubFix;
mean(meanSubFix(:,2))
mean(meanSubFix(:,3))
std(meanSubFix(:,2))
std(meanSubFix(:,3))
[meanSubFixTest,P,CI,STATS] = ttest((meanSubFix(:,2)-meanSubFix(:,3)))
CohenDFix = mean(meanSubFix(:,2)-meanSubFix(:,3))/STATS.sd
%per stim
S=unique(ResProcAdd(:,3)); Sbis=unique(ResProcAdd(:,4));
S=[S;Sbis]; S=unique(S);
for i=1:size(S,1)
    j=S(i);
    LineL=find(ResProcFix(:,3)==j);
    LineR=find(ResProcFix(:,4)==j);
    S1=ResProcFix(LineL,:);
    S2=ResProcFix(LineR,:);
    if (~isempty(LineR) & ~isempty(LineL))
        meanStimFix(i,:)=[j mean([S1(:,9);S2(:,10)]) (dist(1,j))];
    elseif ((isempty(LineL))& isempty(LineR))
```

```
    elseif (isempty(LineL)& ~isempty(LineR))
        meanStimFix(i,:)=[j mean([S2(:,10)]) (dist(1,j))];
    elseif (isempty(LineR)&~isempty(LineL))
        meanStimFix(i,:)=[j mean([S1(:,9)]) (dist(1,j))];
    end
end
figure(3);plot(meanStimFix(:,3),meanStimFix(:,2),'r*')
[rFix,p]=corrcoef(meanStimFix(:,3),meanStimFix(:,2))

% title(strcat('Correlation between fixation number and distance from the
prototype'),'fontsize',14);
%  set(gca,'linewidth',2,'fontsize',14)
%  xlabel('distance from the prototype','fontsize',14);
%  ylabel('mean number of fixations','fontsize',14);

%% analyse by stim
S=unique(ResProcAdd(:,3)); Sbis=unique(ResProcAdd(:,4));
S=[S;Sbis]; S=unique(S);

for i=1:size(S,1)
    j=S(i);
    LineL=find(ResProcAdd(:,3)==j);
    LineR=find(ResProcAdd(:,4)==j);
    S1=ResProcAdd(LineL,:);
    S2=ResProcAdd(LineR,:);
    if (~isempty(LineR) & ~isempty(LineL))
        meanStim(i,:)=[j mean([S1(:,5);S2(:,6)]) (dist(1,j))];
    elseif ((isempty(LineL))& isempty(LineR))
    elseif (isempty(LineL)& ~isempty(LineR))
        meanStim(i,:)=[j mean([S2(:,6)]) (dist(1,j))];
    elseif (isempty(LineR)&~isempty(LineL))
        meanStim(i,:)=[j mean([S1(:,5)]) (dist(1,j))];
    end
end
figure(2);plot(meanStim(:,3),meanStim(:,2),'r*')
[rstim,P,RL,RU]=corrcoef(meanStim(:,3),meanStim(:,2))

% title(strcat('Correlation between looking time and distance from the
prototype'),'fontsize',14);
%  set(gca,'linewidth',2,'fontsize',14)
%  xlabel('distance from the prototype','fontsize',14);
%  ylabel('mean looking time on faces','fontsize',14);

%mean fixation  duration
mean(ResProcFix(:,22))
std(ResProcFix(:,22))
mean(ResProcFix(:,23))
std(ResProcFix(:,23))
[FixResTest,P,CI,STATS] = ttest((ResProcFix(:,22)-ResProcFix(:,23)))
CohenDfixdur = mean(ResProcFix(:,22)-ResProcFix(:,23))/STATS.sd
```

```
%%to make it work:
%%place the Data.xls file in the same folder than the current script.
%%script to run the analyze from:
%%Perception of facial averageness: Evidence for a common mechanism in
%%human and macaque infants
%%%Experiment 2 3 and 4.
%%% depending on the Experiment you want to analyze, don't forget to
%%% comment the line calling the data of the other experiments



clc;clear all;close all;
pathim='.\';
%%Experiment 2:
Res = xlsread('Data.xls','DataMonkInfantExp2','A2:J209');
dist= xlsread('Data.xls','DistanceMonkInfant','A2:B17');
%%For infant in Exp 2: after outlier analysis based on median absolute
%%deviation
Filt5=find(Res(:,1)==32.900000000000000); Res(Filt5,:)=[];%% outlier
Filt5=find(Res(:,1)==24.960000000000000); Res(Filt5,:)=[];%% outlier

%%Experiment 3: (don't forget to comment lines for Experiment 2 above)
%Res = xlsread('Data.xls','DataMonkAdultExp3','A2:J209');
%dist= xlsread('Data.xls','DistanceMonkAdult','A2:B17');


%%For Experiment 4 -adult participants-
% Res = xlsread('Data.xls','DataHumanAdultExp4','A2:J145');
% dist= xlsread('Data.xls','DistanceMonkInfant','A2:B17');

%%For Experiment 4 -infant participants-
%Res = xlsread('Data.xls','DataHumanInfantExp4','A2:J129');
%dist= xlsread('Data.xls','DistanceMonkInfant','A2:B17');


dist=dist';

%%Preprocess: outlier and excuded trials

%% remove trial with o msec of looking time on one side
Filt3=find((Res(:,6))==0);Res(Filt3,:)=[];
Filt3=find((Res(:,5))==0);Res(Filt3,:)=[];
 Filt3=find((Res(:,5)+Res(:,6))==0);Res(Filt3,:)=[];

%%fixation duration
%FixRes=[Res(:,5)./Res(:,7) Res(:,6)./Res(:,8)];

%%%%First: TEst of normality
%  hist(FixRes,20);
 %
NormFix=(((FixRes(:,1)-FixRes(:,2))-(mean((FixRes(:,1)-FixRes(:,2)))))/(std
(FixRes(:,1)-FixRes(:,2))));
 %[H3,P,ksstat] = kstest(NormFix)
%  if H3==0
```

```
%  else
% return
%  end
% %because lt are lognormal distrib
%
logNormFix=(((log(FixRes(:,1))-log(FixRes(:,2)))-(mean((log(FixRes(:,1))-lo
g(FixRes(:,2))))))...
%     /(std(log(FixRes(:,1))-log(FixRes(:,2)))));
% hist(logNormFix,20);
% [H4,P,ksstat] = kstest(logNormFix)
%  if H4==0
%   %%FixRes=[log(Res(:,5)./Res(:,7)) log(Res(:,6)./Res(:,8)) Res(:,9:10)];

%  else
%  end
% %%look at the data
%  hist((Res(:,5)-Res(:,6)),20)
%   hist((Res(:,7)-Res(:,8)),20)
 %%%z score of LT to perfrom KSTEST (normality test)%%%

Norm=(((Res(:,5)-Res(:,6))-(mean((Res(:,5)-Res(:,6)))))/(std(Res(:,5)-Res(:
,6))));
[H,P,ksstat] = kstest(Norm)

Norm2=(((Res(:,7)-Res(:,8))-(mean((Res(:,7)-Res(:,8)))))/(std(Res(:,7)-Res(
:,8))));
 [H2,P,ksstat] = kstest(Norm2)
% if H==0
 %else
%[H,STATS] = CDFPLOT(Norm);
%return
% end
%  if H2==0
%  else
% [H2,STATS] = CDFPLOT(Norm2);
% %return
%  end
% % %because lt are lognormal distrib
%
logNorm=(((log(Res(:,5))-log(Res(:,6)))-(mean((log(Res(:,5))-log(Res(:,6)))
))))...
%     /(std(log(Res(:,5))-log(Res(:,6)))));
% hist(logNorm,20);
% [H,P,ksstat] = kstest(logNorm)
% return
%  if H==0
%  else
% [H,STATS] = CDFPLOT(logNorm);
%  end
%
logNorm2=(((log(Res(:,7))-log(Res(:,8)))-(mean((log(Res(:,7))-log(Res(:,8))
))))...
%     /(std(log(Res(:,7))-log(Res(:,8)))));
% hist(logNorm2,20);
```

```
% [H2,P,ksstat] = kstest(logNorm2)
%  if H2==0
%  else
% [H,STATS] = CDFPLOT(logNorm);
%  end


 %%%%%if normality is not ok: log transform
  FixRes=[log(Res(:,5)./Res(:,7)) log(Res(:,6)./Res(:,8)) Res(:,9:10)];
    Res=[Res(:,1:4) log(Res(:,5)) log(Res(:,6))...
   log(Res(:,7)) log(Res(:,8)) Res(:,9:10)];
%%%%%else
%FixRes=[Res(:,5)./Res(:,7) Res(:,6)./Res(:,8) Res(:,9:10)];


%%%%Now Analyze the data

%%to check the number of trial per subject,
Restemp=Res(:,1)*1000;
T=unique(Restemp);
 for su = 1:size(T,1)
      TestTrial=find(Restemp(:,1)==T(su));
      if isempty(TestTrial)
      else
           Nb(su)=sum([TestTrial./TestTrial]);
      end
 end
      mean(Nb)
      std(Nb)
%calculate the difference between time and between dist
for k=1:size(Res,1)
    DiffTime=(Res(k,5)-(Res(k,6)));
    DiffDist=(Res(k,9)-(Res(k,10)));
    ResProc(k,:)=[Res(k,:) DiffTime DiffDist];
end

%%per subject:
for i = 1:size(ResProc,1)
    if ResProc(i,12)<0
        ResProcAdd(i,:)=[ResProc(i,:) ResProc(i,5) ResProc(i,6)...
            (ResProc(i,5)-ResProc(i,6)) (ResProc(i,9)-ResProc(i,10))];
    else
        ResProcAdd(i,:)=[ResProc(i,:) ResProc(i,6) ResProc(i,5)
(ResProc(i,6)-ResProc(i,5))...
            (ResProc(i,10)-ResProc(i,9))];
    end
end

G=unique(ResProcAdd(:,1));
for i=1:size(G,1)
    Line=find(ResProcAdd(:,1)==G(i));
    G1=ResProcAdd(Line,:);
    meanSub(i,:)=[G(i) mean(G1(:,13)) mean(G1(:,14))];
end
```

```
[meanSubTest,P,CI,STATS] = ttest((meanSub(:,2)-meanSub(:,3)))
CohenDSub = mean(meanSub(:,2)-meanSub(:,3))/STATS.sd
%return
% to calculate the difference in terms of fixation number
for i = 1:size(ResProcAdd,1)
    if ResProcAdd(i,12)<0
        ResProcFix(i,:)=[ResProcAdd(i,:) ResProcAdd(i,7) ResProcAdd(i,8)];
    else
        ResProcFix(i,:)=[ResProcAdd(i,:) ResProcAdd(i,8) ResProcAdd(i,7)];
    end
end

H=unique(ResProcFix(:,1));
for i=1:size(H,1)
    Line=find(ResProcFix(:,1)==H(i));
    H1=ResProcFix(Line,:);
    meanSubFix(i,:)=[H(i) mean(H1(:,17)) mean(H1(:,18))];
end

[meanSubFixTest,P,CI,STATS] = ttest((meanSubFix(:,2)-meanSubFix(:,3)))
CohenDFix = mean(meanSubFix(:,2)-meanSubFix(:,3))/STATS.sd
%per stim
 S=unique(ResProcFix(:,3));
  for i=1:size(S,1)
      j=S(i);
    LineL=find(ResProcFix(:,3)==j);
      LineR=find(ResProcFix(:,4)==j);
    S1=ResProcFix(LineL,:);
    S2=ResProcFix(LineR,:);
    if (isempty(LineL))
    else
 DistanceF =find(dist(2,:)==S(i));
    meanStimFix(i,:)=[j mean([S1(:,7);S2(:,8)]) (dist(1,DistanceF))];
    end
  end
 %% to do a figure
% figure(3);plot(meanStimFix(:,3),meanStimFix(:,2),'r*')
%  [rFix,p] = corrcoef(meanStimFix(:,3),meanStimFix(:,2))
% title(strcat('Correlation between fixation number and distance from the
prototype'),'fontsize',14);
%  set(gca,'linewidth',2,'fontsize',14)
%  xlabel('distance from the prototype','fontsize',14);
%  ylabel('mean number of fixations','fontsize',14);
  %% analyse by stimuli
 S=unique(ResProcAdd(:,3));
  for i=1:size(S,1)
      j=S(i);
    LineL=find(ResProcAdd(:,3)==j);
      LineR=find(ResProcAdd(:,4)==j);
    S1=ResProcAdd(LineL,:);
    S2=ResProcAdd(LineR,:);
    if (isempty(LineL))
    else
```

```
     Distance =find(dist(2,:)==S(i));
    meanStim(i,:)=[j mean([S1(:,5);S2(:,6)]) (dist(1,Distance))];
    end
  end
figure(2);plot(meanStim(:,3),meanStim(:,2),'r*')
[rstim,P,RL,RU]= corrcoef(meanStim(:,3),meanStim(:,2))
% title(strcat('Correlation between looking time and distance from the
prototype'),'fontsize',14);
%  set(gca,'linewidth',2,'fontsize',14)
%  xlabel('distance from the prototype','fontsize',14);
%  ylabel('mean looking time on faces','fontsize',14);
return
%%check the fixation
for k=1:size(FixRes,1)
   % DiffTime=(Res(k,5)-(Res(k,6)));
    Dif=(FixRes(k,3)-(FixRes(k,4)));
    FixResProc(k,:)=[FixRes(k,:) Dif];
end
FixRes=FixResProc;
%%per subject:
for i = 1:size(FixResProc,1)
    if FixResProc(i,5)<0
        FixResProc1(i,:)=[FixResProc(i,:) FixResProc(i,1)
FixResProc(i,2)...
            (FixResProc(i,1)-FixResProc(i,2))
(FixResProc(i,3)-FixResProc(i,4))];
    else
        FixResProc1(i,:)=[FixResProc(i,:) FixResProc(i,2) FixResProc(i,1)
(FixResProc(i,2)-FixResProc(i,1))...
            (FixResProc(i,4)-FixResProc(i,3))];
    end
end
FixRes=FixResProc1(:,6:7);
mean(FixRes(:,1))
mean(FixRes(:,2))
std(FixRes(:,1))
std(FixRes(:,2))
[FixResTest,P,CI,STATS] = ttest((FixRes(:,1)-FixRes(:,2)))
CohenDfixdur = mean(FixRes(:,1)-FixRes(:,2))/STATS.sd
```
